# Enhanced tumour antiangiogenic effects when combining gefitinib with the antivascular agent ZD6126

**DOI:** 10.1038/sj.bjc.6603308

**Published:** 2006-08-29

**Authors:** A Bozec, S Lassalle, J Gugenheim, J-L Fischel, P Formento, P Hofman, G Milano

**Affiliations:** 1 Oncopharmacology Unit, Centre Antoine-Lacassagne, 33, Avenue de Valombrose, 06189 Nice Cedex 2, France; 2 General Hospital, Nice, France

**Keywords:** EGFR targeting, antivascular agents, gefitinib, ZD6126

## Abstract

Current experimental and clinical knowledge supports the optimisation of endothelial cell targeting using a strategy combining anti-EGFR drugs with antivascular agents. The purpose of the present study was to examine the effects of the association of ZD6126, an antivascular microtubule-destabilising agent, with gefitinib and irradiation on the growth of six head and neck human cancer cell lines xenografted in nude mice and to study predictive and molecular factors responsible for antitumour effects. CAL33- and Hep-2-grafted cell lines were the most sensitive to ZD6126 treatment, with VEGF levels significantly higher (*P*=0.0336) in these tumour xenografts compared to Detroit 562- and CAL27-grafted cell lines with relatively low VEGF levels that were not sensitive to ZD6126. In contrast, neither IL8 levels nor EGFR expression was linked to the antitumour effects of ZD6126. ZD6126 in combination with gefitinib resulted in a synergistic cytotoxic interaction with greater antitumour effects than gefitinib alone. The synergistic interaction between ZD6126 and gefitinib was corroborated by a significant decrease in CD31 labelling. The present study may serve for future innovative clinical applications, as it suggests that VEGF tumour levels are possible predictors for ZD6126 antitumour efficacy. It also supports the notion of antitumour supra-additivity when combining gefitinib and ZD6126, and identifies neoangiogenesis as the main determinant of this synergistic combination.

Tumour expression of growth factor receptors of the HER family makes it possible to perform innovative therapeutic targeting as demonstrated by the development of clinically active drugs such as herceptin(®), cetuximab and EGFR tyrosine kinase inhibitors (Gross *et al*, 2004). The antiproliferative and antitumour effects of these compounds are attributed to cellular physiological consequences following receptor targeting manifested by inhibition of cell proliferation, increased apoptosis and reduced tumour neoangiogenesis (Woodburn, 1999). This ability to reduce tumour neoangiogenesis by EGFR targeting has been attributed to the inhibition of proangiogenic tumour factors such as VEGF and FGF (Perrotte *et al*, 1999; Ciardiello *et al*, 2001). An interesting recent study points out that EGFR targeting of tumours can also include the endothelial cell network (Hirata *et al*, 2002). Endothelial cells express EGFR. Thus, part of the global antiangiogenic effect resulting from EGFR targeting can also be attributed to the direct impact of EGFR-targeting agents on endothelial cells. Consequently, a variable part of the antiangiogenic activity of EGFR-targeting drugs could be caused by a dual effect: inhibition of proangiogenic factors such as VEGF produced by the tumour itself and a direct effect on the intra-tumour endothelial cells. It would be interesting therefore to optimise endothelial cell targeting using a strategy combining anti-EGFR treatment and an antivascular approach. The vascular-targeting compound ZD6126 destabilises microtubules and selectively disrupts immature tumour endothelial cells, which stops tumour blood flow and induces tumour cell death (Thorpe *et al*, 2003). A recent phase I study with ZD6126 underlines the potential impact of this compound on the vasculature (Beerepoot *et al*, 2006). Associating ZD6126 with the EGFR tyrosine kinase inhibitor gefitinib would thus combine an antivascular agent and an anti-EGFR drug. Furthermore, as ZD6126 is a compound acting on microtubules, it cannot be excluded that part of its antitumour activity may also consist of direct effects on tumour cells. Combining ZD6126 and gefitinib would thus provide a potentially beneficial association of two drugs producing optimal effects on both endothelial and tumour cells. Recent preclinical studies suggest that radiotherapy in combination with antiangiogenic/vasculature-targeting agents may enhance the therapeutic ratio of ionising radiation alone (Wachsberger *et al*, 2005). Thus, the association of ZD6126 and gefitinib with irradiation could also be an interesting area for investigation.

Tumour vasculature is a key target in the treatment of solid tumours, particularly of the head and neck (Le and Giaccia, 2003). In the present study, a panel of six different head and neck human cancer cell lines was examined in a xenograft mouse model. These tumour xenografts, in athymic nude mice, were studied for predictive and molecular factors responsible for antitumour effects.

## MATERIALS AND METHODS

### Chemicals

Gefitinib and ZD6126 were kindly provided by AstraZeneca (Macclesfield, United Kingdom). Working solutions were prepared extemporaneously as follows: gefitinib was suspended in 0.9% NaCl, 0.01% Tween-80 and ZD6126 in 0.9% NaCl with pH adjusted to 4.5. For both drugs, the concentrations were adjusted so as to include the daily dose in 0.2 ml of drug suspension. Dulbecco's modified Eagle's medium (DMEM), penicillin, streptomycin and glutamine were purchased from Whittaker (Verviers, Belgium). Foetal bovine serum (FBS) was obtained from Dutscher (Brumath, France).

### Cell lines

Six different human head and neck cancer cell lines: CAL27, CAL33, CAL60 and CAL166 originated from our institution (the Centre Antoine Lacassagne); Detroit 562 and Hep-2 were obtained from the American Type Culture Collection (Rockville, MD, USA). These cell lines exhibit variable EGFR levels (ligand-binding assay; Dassonville *et al*, 1993), doubling time, *in vitro* sensitivities to gefitinib or RT and p53 status, as summarised in Table 1.

The cell lines were maintained as monolayer cultures in DMEM supplemented with 10% FBS (v v^−1^), 2 mM glutamic acid, 50 000 U l^−1^ penicillin and 80 *μ*M streptomycin in a humidified incubator (Sanyo, Japan) at 37°C in an atmosphere containing 8% CO_2_. Batches of 15 × 10^6^ cells were frozen in FBS supplemented with 5% DMSO (v v^−1^) in advance for injection into mice. Shortly before injection, cells were thawed and suspended in Ringer lactate.

### Mice

Animal experiments were performed in accordance with the regulations of the institutional ethical commission and of the United Kingdom Co-ordinating Committee on Cancer Research guidelines (Workman *et al*, 1998). Six-week-old female Swiss nude mice were purchased from Charles River (L'Arbresle, France) and received subcutaneous inoculation in the right flank of 2 × 10^6^ cells dissolved in 100 *μ*l of Ringer lactate (six to eight animals per treatment condition).

### Tumours

Tumour length and width were measured weekly using a caliper. Tumour volume was calculated as *π*/6 × length × width^2^ until animal killing. At that time, animals were killed by spinal cord dislocation and tumours were subsequently removed surgically and weighed; half of the tumour was directly frozen in liquid nitrogen for protein analysis and the other half fixed in para-formaldehyde overnight for CD31 and Ki67 examination using microtissue array (MTA immunochemistry).

### Preparation of samples for analysis of cellular factors

Frozen tumours were pulverised in a liquid nitrogen-cooled Thermovac pulveriser. The resulting powders were homogenised in 10 volumes of a 10 mM Tris-HCl buffer (pH 7.4), containing 1 mM EDTA, 0.5 mM dithiothreitol and 10 mM sodium molybdate. The homogenates were centrifuged for 1 h at 105 000 g (+4°C) and the supernatants (cytosols) were used for protein determination by immunoblotting. Total protein content was measured using the bicinchoninic acid assay.

VEGF and IL8 were determined by ELISA using DVE 00 and D8050, respectively, from Quantikine (Minneapolis, USA); EGFR was determined by the ^125^I-EGF binding method followed by Scatchard-plot analysis. These factors were chosen because VEGF is the main growth factor for endothelial cells; IL8 is a proangiogenic factor (as VEGF, bFGF and aFGF) with a proven role in different tumour types and EGFR as it is the target of gefitinib.

The microvessel marker CD31 (DAKO monoclonal antibody M/B1 ref.: M7240) and the proliferation marker Ki67 (DAKO monoclonal antibody JC70A ref.: M0823) were determined by MTA on treated tumours obtained after animal killing.

### Treatment

#### *In vivo* growth characteristics of the human head and neck cancer xenografts

Tumour volume was monitored once a week between 3 and 8 weeks depending on the cell line; the experiments were stopped when tumours reached 1 cm^3^. The differential expression of molecular factors potentially related to ZD6126 sensitivity such as VEGF, IL8 (by ELISA) and EGFR (by ligand-binding assay) was measured, at the end of the observation period, in untreated tumours (tumour volume between 72 and 2344 mm^3^) after animal killing.

#### Sensitivity of the six cell lines to ZD6126

Treatment was applied when tumours reached a mean volume of 250 mm^3^; animals were treated once a week for 3 weeks, with freshly prepared ZD6126 (200 mg kg^−1^) (Goto *et al*, 2004), or vehicle only (controls), and the tumour volume monitored for 2 weeks after the end of treatment period or until the mean volume of the controls reached 1 cm^3^. Mice injected with CAL166 and CAL33 tumours, which grow relatively quickly, could only be treated twice whereas all other mice models were treated three times.

#### Effects of a single drug and their combination with irradiation on tumour growth, tumour vessel density and molecular factors representative of proliferation

The effects of each single drug and their combination with or without irradiation (RT) were examined for the impact on tumour growth and tumour angiogenesis (CD31) and on molecular factors indicative of proliferation (Ki67). CD31 and Ki67 expressions were determined in MTAs by immunohistochemistry (Simon *et al*, 2004) on CAL33, a highly proliferative and moderately responsive cell line. The final score was the result of the examination of three fields per tumour, and between four and eight tumours were investigated. Labelling intensities were scored as 0=no, 1=slight, 2=medium, 3=strong and 4=very strong. Scoring was performed at the end of the treatment sequence, on day 15, and at the end of experiments on day 33. Time of termination of experiments was dictated by ethical reasons (tumour volume in controls).

Mice bearing well-established CAL33 tumours (mean tumour volume/treatment group ∼250 mm^3^) were treated every week with vehicle alone (controls), ZD6126 (150 mg kg^−1^, 0.2 ml intraperitoneally on days 0, 7 and 14), gefitinib (120 mg kg^−1^ day^−1^, 5 days week^−1^ during 2 weeks, 0.2 ml os^−1^) and RT (3 Gy day^−1^, 3 days week^−1^ during 2 weeks). The dose of gefitinib was chosen according to previous data in tumour xenografts in mice (Formento *et al*, 2005) and the dose of ZD6126 was diminished when compared to the monotherapy study in order to allow the interaction with other agents to be put into evidence.

Interaction between ZD6126 and gefitinib alone or in combination with RT (when given on the same day, ZD6126 was applied 2 h after RT) was evaluated for tumour growth inhibition. The sequence between gefitinib and RT was already established by us from tumour experiments with the two agents applied to the same tumour cell line xenograft (Formento *et al*, 2005).

The effects of the treatments were evaluated as described previously (Prewett *et al*, 2002). Evaluation of the tumour effect consisted in measuring the mean tumour volume on different given days for the different treatment groups: controls, treatment a, treatment b and treatment a+b. Fractional tumour volume (FTV) for each treatment group was calculated as the ratio between the mean tumour volumes of treated and untreated tumours. This was performed for treatment a (FTVa), for treatment b (FTVb) and for treatment a+b (FTVa+b). The expected FTV for the ‘a+b’ combination was defined as FTVa (observed) × FTVb (observed). The ratio FTVa+b (expected)/FTVa+b (observed) was the combination ratio (CR). If CR >1, there are supra-additive effects and if CR <1 infra-additive ones. Strictly additive effects are observed if CR=1.

The effects on CD31 and Ki67 were evaluated using the non-parametric analysis of variance (Kruskal–Wallis test). The differences between cell lines for molecular factors (VEGF, IL8 and EGFR) were examined using the Mann–Whitney test.

## RESULTS

### Tumour characteristics

**Figure 1:**
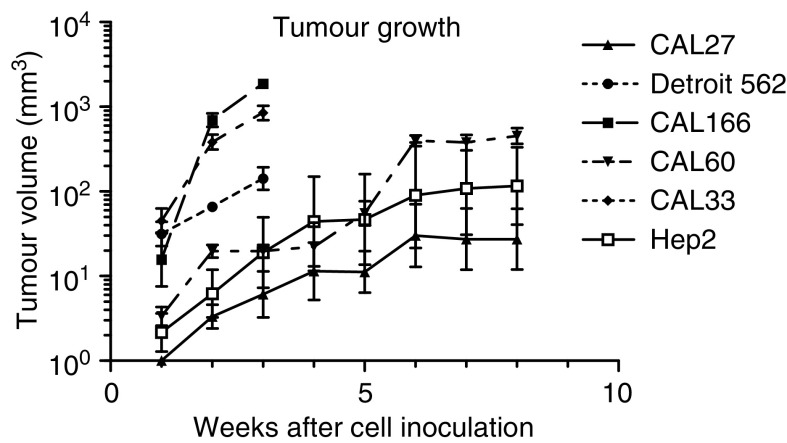
Growth (log scale) of the different xenografted tumours in athymic nude mice. Bars represent standard error of the mean.

The fastest growing xenografts were CAL166 and CAL33 with a tumour volume doubling time of 7 and 10 days, respectively, followed by Detroit 562 (tumour volume doubling time of 15 days) (Figure 1). The other cell lines (CAL60, Hep-2 and CAL27) grew relatively more slowly under the conditions of the present study.

**Figure 2:**
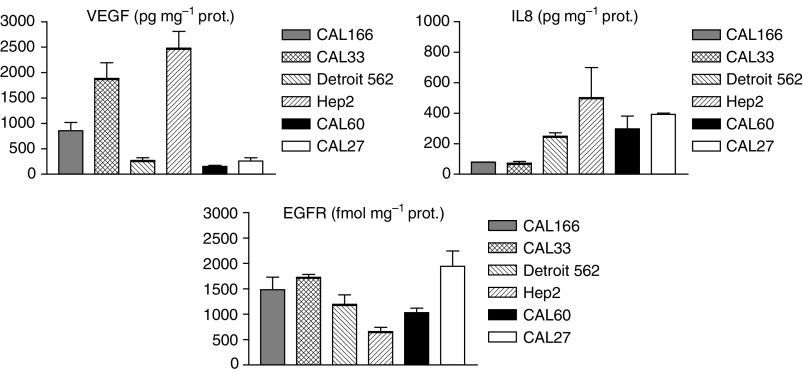
Distribution of VEGF (pg mg^−1^ protein), IL8 (pg mg^−1^ protein) and EGFR (fmol mg^−1^ protein) levels in different xenografted tumours in athymic nude mice. Bars represent standard error of the mean.

There was a marked variation in VEGF and IL8 levels between the six xenograft models examined. The variability in EGFR expression was smaller between the studied cell lines (Figure 2).

**Figure 3:**
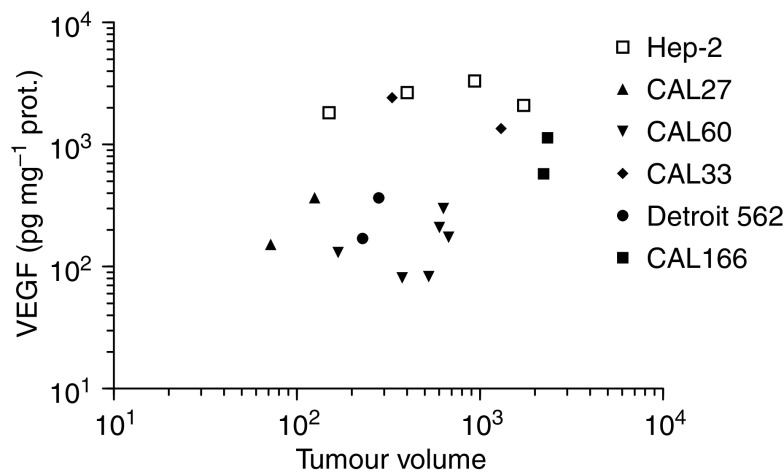
Distribution of individual tumour VEGF levels as a function of tumour volume. A log–log scale was adopted due to the marked variations in the *x*- and *y*-axis.

The tumour volume did not influence VEGF secretion (Figure 3); tumours originating from the same cell line, whatever their respective volumes, maintain very comparable VEGF levels. The VEGF intra-tumour concentration could thus be considered to be strictly cell line dependent.

### Effects of single-agent ZD6126

**Figure 4:**
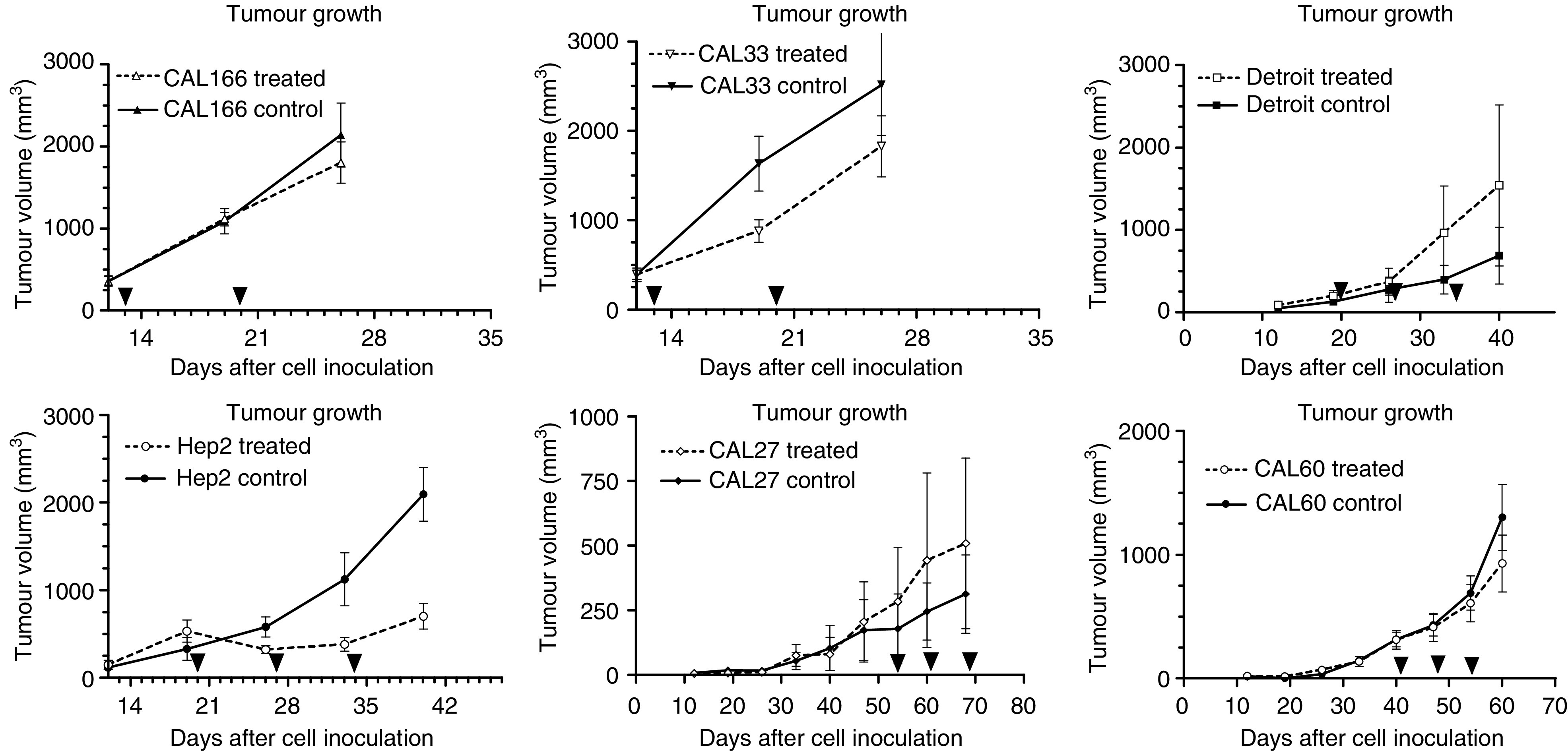
Effects of ZD6126 against CAL33 human head and neck xenografts (filled triangles indicate the time of treatment). Bars represent standard error of the mean.

ZD6126 on its own exhibited relatively modest tumour growth inhibition. There were marked disparities in the antitumour activity of ZD6126 between the different xenografts (Figure 4). Of note, for Detroit and CAL27 tumour xenografts, treatment with ZD6126 conferred a growth advantage. CAL33 was moderately sensitive and Hep-2 was the most sensitive to ZD6126 treatment. VEGF levels were significantly higher (*P*=0.0336) in these tumour xenografts as compared to Detroit 562 and CAL27 xenografts for which ZD6126 increased the tumour growth (Figure 2). Neither EGFR nor IL8 tumour levels were linked to the differential sensitivity to ZD6126.

### Effects of ZD6126 in combination with gefitinib and radiation

**Figure 5:**
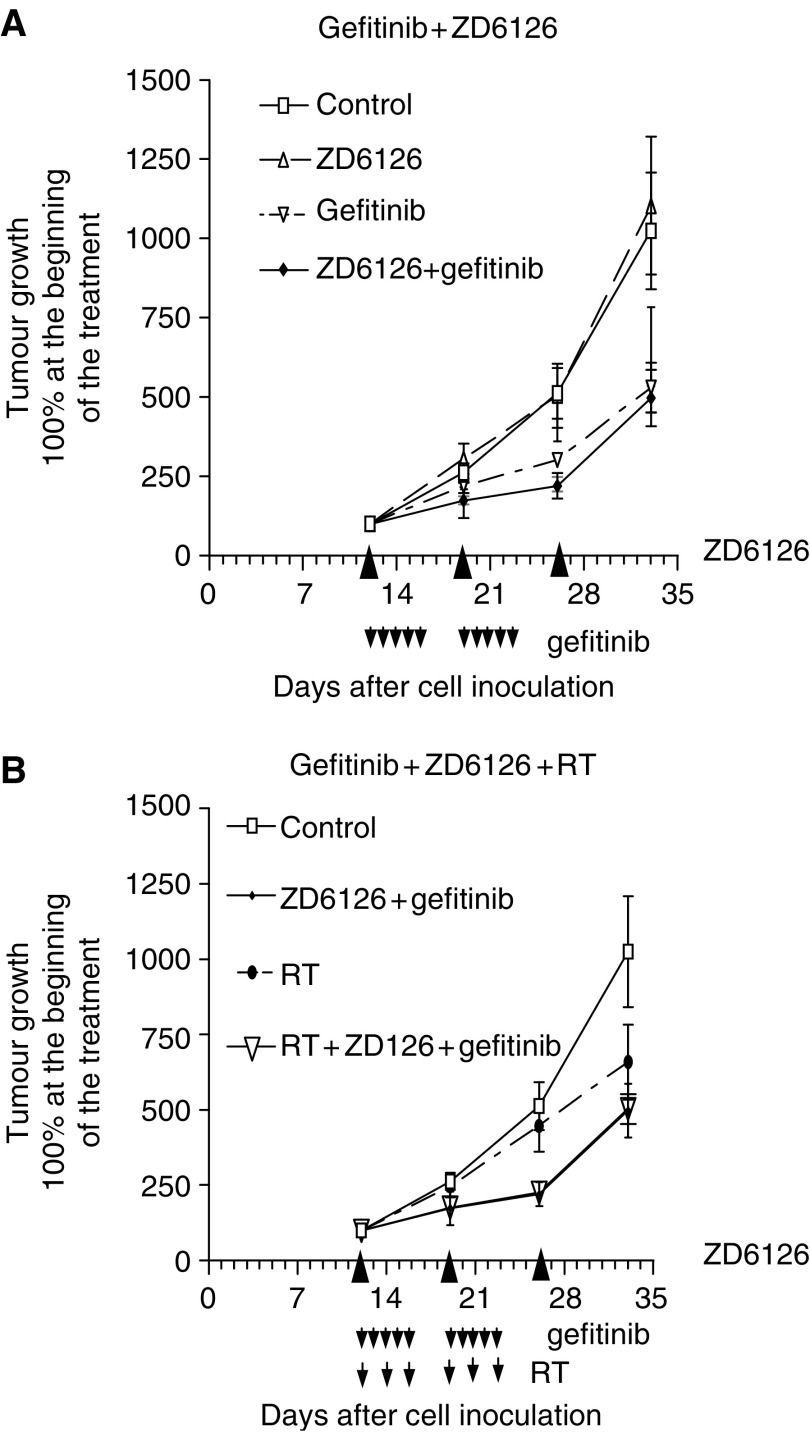
(**A**) Effects of ZD6126 and gefitinib or ZD6126+gefitinib on CAL33 tumour xenografts (filled triangles and arrows indicate the time of treatments). Bars represent standard error of the mean. (**B**) Effects of ZD6126+gefitinib+RT on CAL33 tumour xenografts (filled triangles and arrows indicate the time of treatments). Tumour growth curves of ZD6126+gefitinib and ZD6126+gefitinib+RT were superimposed. Bars represent standard error of the mean.

Figure 5A shows that, although ZD6126 had no apparent effect on CAL33 tumour growth, its combination with gefitinib resulted in a greater antitumour effect than with gefitinib alone (CR=1.47, 1.35 and 1.15 at 19, 26 and 33 days, respectively, after cell injection, with between five and eight mice per treatment group). The addition of RT did not modify markedly the observed antitumour effects resulting from the ZD6126+gefitinib combination with a tendency in this case to infra-additive interaction (CR=0.87, 0.85 and 0.64 at 19, 26 and 33 days, respectively, after cell injection) (Figure 5B).

### Molecular parameters

**Figure 6:**
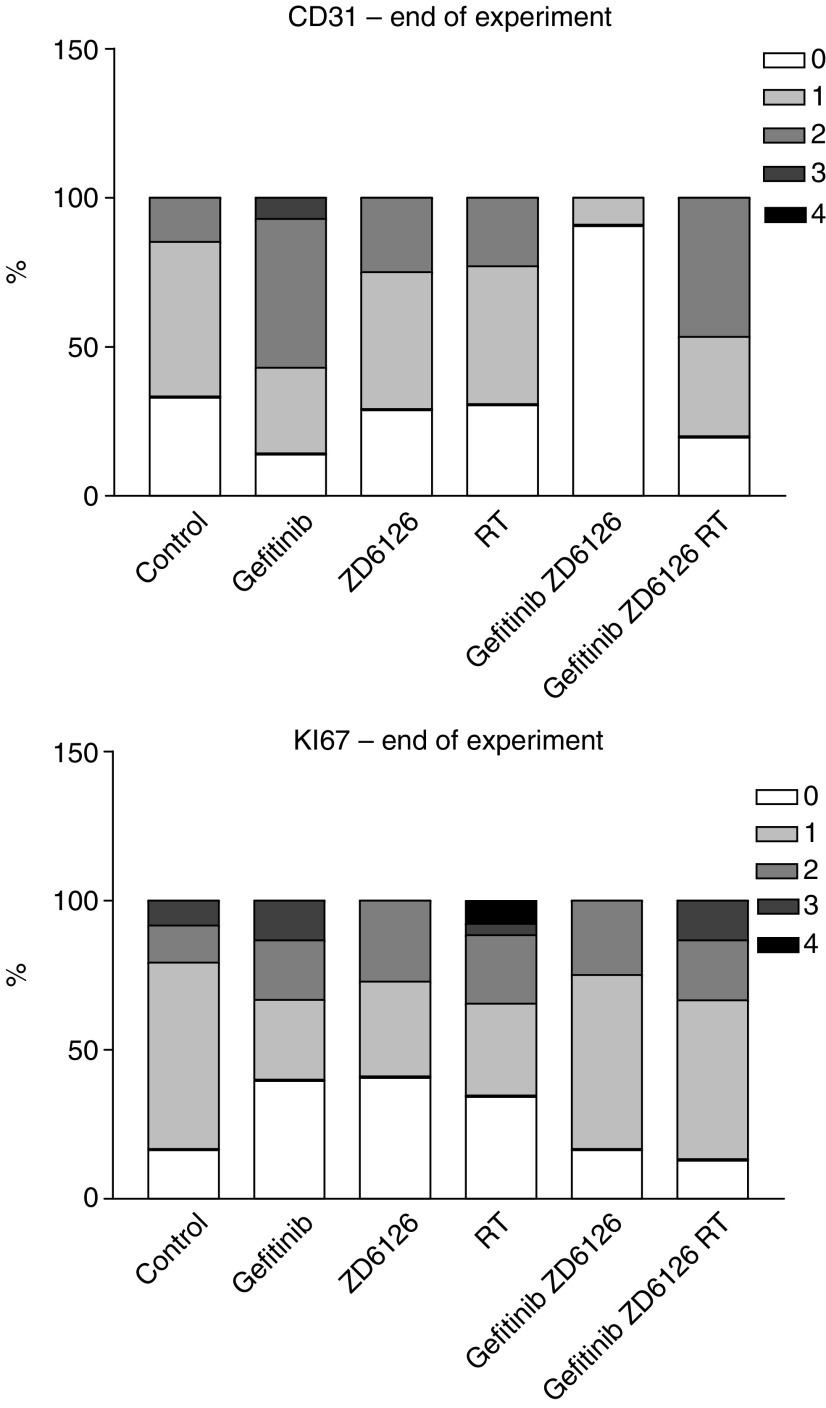
Effects of ZD6126, gefitinib, RT, ZD6126+gefitinib and ZD6126+gefitinib+RT on the labelling frequency and intensity of CD31 (endothelial cell marker) and Ki67 (proliferation marker) in CAL33 tumour xenografts. The final score was the result of the examination of three fields per tumour, and between four and eight tumours were investigated. Labelling intensities are shown as 0=no, 1=slight, 2=medium, 3=strong and 4=very strong, with the sum of all values in one histogram being 100%. Scoring was performed at the end of experiments on day 33.

At the end of the experiment (day 33), CAL33 tumours were analysed for CD31 and Ki67 staining. Single-drug treatments with either gefitinib or ZD6126 had a modest effect on CD31 tumour labelling as compared to controls without drug (Figure 6). CD31 tumour labelling reflected accurately the antitumour effect resulting from the different combinations. Thus, the synergistic interaction between ZD6126 and gefitinib was confirmed by a significant decrease in CD31 labelling (*P*<0.01) (Figure 6). There was a less intense impact on CD31 labelling, corroborating the effects on tumour growth, when applying the triple combination ZD6126+gefitinib+RT as compared to the two-drug association (Figure 6). In contrast to what was observed with CD31 labelling, there was no clear link between Ki67 labelling and drug effects (Figure 6). Similar observations were made at the end of the treatment (day 15), but the relative diminution in CD31 labelling resulting from the ZD6126–gefitinib association was less marked at this time than at the end of the experiment on day 33 (data not shown).

## DISCUSSION

Tumour neoangiogenesis is a complex process that involves multiple and interrelated steps dependent on positive and negative regulatory growth factors (Wray *et al*, 2004). Given its importance in the development of tumour-associated neoangiogenesis (Ferrara, 2005), the VEGF pathway has received constant attention as a target in antiangiogenesis strategies using various approaches (Ferrara, 2004). Among these approaches, one of the most rewarding approach so far at the clinical level is the use of the monoclonal antibody bevacizumab, which acts directly by depleting VEGF levels in physiological fluids (Stern and Herrmann, 2005). Another complementary tumour antiangiogenic strategy consists in developing agents that are able to act directly on the function of established endothelial cells in the tumour (Thorpe *et al*, 2003). Microtubule-destabilising agents belong to this category, where their mechanism of action is focused on the disruption of the endothelial cell cytoskeleton (Hsieh *et al*, 2005). One of the drugs belonging to this therapeutic class is ZD6126, a phosphate prodrug of *N*-acetyl colchinol which has been shown to induce pronounced but reversible changes in immature endothelial cell morphology and to have marked vascular effects and antitumour activity in preclinical models (Davis *et al*, 2002). Antiangiogenic and vascular disrupting therapies target, at different levels, the expanding tumour endothelial cell network. It was tempting, therefore, to combine these classes of drugs. Such an approach was recently adopted by Siemann and Shi (2004). The authors report that significant antitumour efficacy could be achieved by associating the potent VEGF-R2 and EGFR inhibitor ZD6474 with ZD6126. As endothelial cells express EGFR, some of the global antiangiogenic effects resulting from the use of EGFR-targeting drugs may also be attributed to the direct impact on endothelial cells (Hirata *et al*, 2002). Using the same strategy, associating different antiangiogenic approaches, the present study combined the well-known anti-EGFR drug gefitinib with the antivascular agent ZD6126. The study focused on human head and neck cancer cell lines as neoangiogenesis plays a critical role in the development and evolution of tumours arising from the upper aero-digestive tract (Nakaya *et al*, 2005; Shang and Lir, 2005). Head and neck cancer is also known to express high levels of EGFR with a strong prognostic value, and the ‘drugable’ relevance of this growth factor has been reported by others (Pomerantz and Grandis, 2003, 2004) and us (Dassonville *et al*, 1993; Etienne *et al*, 1999; Pivot *et al*, 2005).

There is evidence that ZD6126 is active in various preclinical tumour models with variable antitumour efficacy (Blakey *et al*, 2002; Evelhoch *et al*, 2004; Skliarenko *et al*, 2006). One of the objectives of the present investigation was to examine the existence of predictive intra-tumour factors that may explain the observed effects. Based on the study of a panel of six different human cancer cell lines of head and neck origin, it was confirmed that treatment with ZD6126 results in variable antitumour effects. Of note, from the study by Skliarenko *et al* (2006), there were tumours for which the application of ZD6126 resulted in greater tumour growth as compared to controls. This phenomenon of tumour re-growth corroborates the present observation of higher growth than in controls for Detroit and CAL27 xenograft under treatment by ZD6126. Antitumour efficacy was observed for CAL33 and Hep-2 cell lines. Interestingly, these two cell lines were those for which established tumours in animals expressed the highest VEGF levels. This result is not particularly surprising and could be explained, as a proof of the concept, by the fact that tumours with a high expression of VEGF may be more dependent on neoangiogenesis and the most sensitive to an antivascular therapeutic approach with ZD6126. Neither EGFR nor IL8 levels were associated with the differences in antitumour effects of ZD6126. The study by Skliarenko *et al* (2006) put into evidence that tumours with higher initial interstitial fluid pressure showed enhanced cell survival following treatment with ZD6126. Thus, intrinsic tumour angiogenesis may be related to the antitumour efficacy of ZD6126. These findings may be useful at the clinical level as there is a risk of a tumour-promoting effect of ZD6126. Selection of appropriate candidates for treatment seems mandatory and could be based on intra-tumour expression of VEGF.

The second part of this study was designed in a way similar to the work recently published by Raben *et al* (2004) who combined ZD1839 (gefitinib) with ZD6126 and irradiation. The authors reported that the triple association applied to the A549 human non-small-cell lung cancer xenograft model induced the greatest effects on tumour growth and angiogenesis. The conclusions of the present study are somewhat different. First, it is interesting to note that, when examining the gefitinib–ZD6126 association on the CAL33 head and neck human cancer cell line xenograft, it appears that although ZD6126 shows no apparent antitumour efficacy by itself at the dose used in the combination experiment (150 mg per day), the final effects become supra-additive when combined with gefitinib (Figure 5A). This observation was strengthened by the analysis of the impact of treatment on tumour neoangiogenesis (CD31 labelling). Gefitinib or ZD6126 by themselves had no effect on CD31 tumour labelling compared to controls without drug. In contrast, the combination of these two drugs markedly reduced the intensity of CD31 labelling in the tumours (Figure 6). There was in contrast no evidence for an explanation of the supra-additive effects between the two drugs when examining the impact of combined treatment on tumour intrinsic proliferation capacity (Ki67 labelling). Thus, it seems that the beneficial antitumour effect of associating gefitinib and ZD6126 is more related to the targeting of endothelial cells than to a diminution of the intrinsic tumour growth. The mechanistic explanation for this synergistic effect on tumour angiogenesis may lie in the fact that each drug has a distinctive impact on endothelial cells. ZD6126 directly affects the internal structure of the endothelial cell, whereas gefitinib acts through inhibition of EGFR signalling of endothelial cells and by reduced production of proangiogenic factors by tumour cells (Hirata *et al*, 2002). The potential direct impact of ZD6126 on the vasculature has been underlined during a recent phase I study with this compound (Beerepoot *et al*, 2006). Thus, the multiple complementary impacts on endothelial cells may lead to measurable effects on tumour growth, although the effect of ZD6126 alone may not be macroscopically visible at this dose.

Previous experimental studies showed potential beneficial antitumour effects when combining ZD6126 with RT (Siemann and Rojiani, 2002; Raben *et al*, 2004). A recent study (Wachsberger *et al*, 2005), however, drew more contrasting conclusions with data suggesting that the optimal therapeutic benefit of ZD6126 plus RT (U87 glioblastoma xenograft) is schedule-dependent with combinations of ZD6126 before each dose of RT being less effective than RT alone. The importance of the scheduling involving ZD6126 and RT was also reported by a previous study (Siemann and Rojiani, 2002) that showed that this association increased tumour cell killing of KHT mouse sarcoma when given 24 h before RT or 1 h or more following RT, but was not found to be as effective if given 1 h before RT. In the present study, the sequence of association between RT and ZD6126 was taken into consideration as the drug was given 2 h after RT. The impact of the association of RT with drugs was examined with the triple combination and it was found, in the present conditions, that RT did not markedly modify the antitumour effect resulting from the ZD6126–gefitinib association (Figure 5B). Of note, this observation was corroborated by examining CD31 tumour labelling, which showed a less intense impact on this parameter when applying the triple combination as compared to the gefitinib–ZD6126 association.

Overall, the present study may contribute to future innovative clinical applications and suggests that the VEGF tumour level is a possible predictor for ZD6126 antitumour efficacy, that it strengthens the notion of antitumour supra-additive effects when combining gefitinib and ZD6126, identifies neoangiogenesis as the main impact of this synergistic combination and gives no firm support to the benefit of adding RT to the drug association.

## Figures and Tables

**Table 1:** Characteristics of head and neck cancer cell lines (historical data from *in vitro* experiments Magné *et al*, 2002)

**Cell line**	**Origin**	**EGFR level (fmol mg^−1^ protein)**	**p53 status**	**Doubling time (h)**	**IC_50_ radiation (Gy)**	**IC_50_ gefitinib (*μ*M)**
CAL27	CAL	8258 (311)	Mutant (exon 6)	18.2 (1.5)	6.2 (1.1)	17.5 (2.4)
CAL33	CAL	33 794 (624)	Mutant (exon 5)	17.5 (0.1)	2.6 (0.4)	6.07 (0.8)
CAL60	CAL	2703 (101)	Mutant (exon 7)	11.8 (0.2)	5.1 (0.1)	11.4 (0.9)
CAL166	CAL	3253 (126)	Wild type	13.1 (1.0)	6.2 (0.8)	22.8 (4.8)
Hep-2	ATCC	388 (27)	Wild type	10.9 (1.0)	9.8 (0.3)	31.1 (2.4)
Detroit562	ATCC	668 (45)	Mutant (exon 5)	14.8 (0.4)	7.7 (0.1)	20.6 (1.5)

ATCC=American Type Culture Collection, Rockville; CAL=Centre Antoine Lacassagne; EGFR=epidermal growth factor receptor.

Mean values (standard deviation) for EGFR levels, doubling time and IC_50_ values. At least two separate experiments were performed to evaluate the EGFR content, and three individual experiments for the other determinations.
